# Editorial: Women in science: life-course epidemiology and social inequalities in health 2022

**DOI:** 10.3389/fpubh.2023.1202171

**Published:** 2023-06-08

**Authors:** MinJae Lee

**Affiliations:** ^1^Peter O'Donnell Jr. School of Public Health, University of Texas Southwestern, Dallas, TX, United States; ^2^Harold C. Simmons Comprehensive Cancer Center, University of Texas Southwestern, Dallas, TX, United States

**Keywords:** women, Science Technology Engineering Mathematics (STEM), public health, social inequalities, disparities in health, social determinants, diversity equity and inclusion (DEI)

The proportion of women and men in science, technology, engineering, and mathematics (STEM) at undergraduate levels is relatively equal. However, there is a lack of representation of women in senior positions in Public Health. According to the State of U.S. Science and Engineering (2022) by *Science and Engineering Indicators*, women in the United States made up only a third (34%) of those employed in STEM occupations in 2019. As the world continues to struggle with critical issues including the pandemic, global warming/climate change, and social problems, the equal participation and leadership of women in STEM disciplines become more imperative than ever. In the field of Life-Course Epidemiology and Social Inequalities in Health, there are many highly influential and successful women who are contributing to the field and tackling important questions. Yet, women are still underrepresented in various aspects of academic life including authorship in peer-reviewed journals, leadership role in academic programs/organizations (1–3). Women's scientific contributions are systematically less likely to be recognized/credited compared to their male counterparts at the same career stage (4). Several initiatives have been recently created to increase the visibility of women in science (e.g., awards for women in STEM). However, evidence indicates that a gender bias is still present throughout many scientific disciplines.

This Research Topic titled “*Women in Science: Life-Course Epidemiology and Social Inequalities in Health 2022*” aims to highlight outstanding female researchers and their contributions to Public Health, specifically in the field of Life-Course Epidemiology and Social Inequalities in Health. In this collection, we accepted and published four original research studies, one brief research report, one systematic review, and one perspective article, led by women researchers contributing to various fields of research conducted in diverse countries including France, Chile, India, the United States. These seven publications, which could be classified into three themes, i.e., *prevention, care*, and *intervention*, discussed social inequalities, disparities, social determinants, diversity, equity, and inclusion in public health. The following is a summary of the major findings from each of these published studies.

## Prevention

In the first article of this Research Topic, Suárez-Reyes and Fernández-Verdejo compare moderate-vigorous physical activity (MVPA) between adult men and women in Chile and assess the relative contribution of each MVPA domain to total MVPA. To dissect the sex gap in MVPA in the adults of Chile, the authors identified where the differences in MVPA lay in a context-specific manner. Their findings indicate that women had lower MVPA for work/household, for transport, and for leisure domains compared to men. Among those with >0 metabolic equivalents of task (MET) × min/week of MVPA, women had lower mean relative contributions to total MVPA from work/household and leisure domains, but higher from the transport domain. The authors emphasize that public health strategies should promote MVPA in women in the work/household, transport, and leisure domains, and further suggest that various strategies (to break job stereotypes, increase opportunities for leisure, ease active transport, re-design urban environments, and ensure access to education) are required to encourage MVPA in women.

The second article from Zhang et al. describes the status of triple negative breast cancer (TNBC), which is an aggressive subtype resistant to conventional treatments with a poorer prognosis, and the temporal changes of its incidence rate in the US. The data of women diagnosed with breast cancer during 2011–2019 were collected from the National Program of Cancer Registries (NPCR) and Surveillance, Epidemiology and End Results (SEER) Program SEER^*^Stat Database. Among total of 238,848 (or 8.8%) TNBC women that were diagnosed during the study period, the authors found that TNBC occurred disproportionally higher in women of Non-Hispanic Black, younger ages, with cancer at a distant stage or poorly/undifferentiated. Non-Hispanic White and Hispanic TNBC women (~34 years), and Non-Hispanic Black women (≥70 years) during the entire period, as well as Asian or Pacific Islander women in the South region had increased trends between 2011 and 2017. These findings in the incidence rate and its temporal change reflect disparities among age groups, race, regions, and disease stages. The authors suggest that special attention is required to address disparities in disease burden among TNBC women.

The third article published as a brief research report by Rajkumari et al. examines and identifies predictors/determinants of hysterectomy incidence in a cohort of North Indian women. The findings from the study population of 702 women indicate overall hysterectomy incidence rate of 11.59 per 1,000 women-years, which was also similar among pre- and post-menopausal women. In addition, while late age at menarche was negatively associated with incident hysterectomy, folate repletion and high triglyceride (TG) at the baseline were positively associated. The authors emphasize that a high incidence rate of hysterectomy points toward the huge burden of gynecological morbidity and the unavailability of non-invasive protocols, and that such a situation warrants immediate policy intervention in order to address the misuse of hysterectomy. Further, the authors suggest that maintaining TG and folate within normal physiological ranges is beneficial in gynecological ailments necessitating hysterectomy.

## Care

The fourth article in this Research Topic, a case study conducted by Steinman et al., uses existing data to learn how home-based collaborative care model (CCM) was adapted by and for community health workers/promotores (CHWs/Ps) to reduce health inequities in late-life depression and depression care among socially disadvantaged older Latino immigrants. The authors evaluated the implementation and effectiveness of the Program to Encourage Active, Rewarding Lives (PEARLS), a home-based CCM, and found that PEARLS was acceptable, feasible and delivered with fidelity. PEARLS participants had significant reductions in depression severity at 5 months and received support for 2.6 social needs on average. These findings demonstrate the value of engaging CHWs/Ps at community-based social service. Further, the community-based organizations (CBOs) and CHWs/Ps strong trust and rapport, addressing social and health needs alongside depression care, and regular internal and external coaching and consultation, appeared to drive successful implementation and effectiveness. The authors highlight that providing culturally and linguistically appropriate care through trusted providers in accessible settings was essential for improving program fit and impact on depression and upstream social outcomes.

In the fifth article, Richard et al. identify social determinants that are associated with inadequate prenatal care utilization (PCU) in sheltered homeless mothers in the Greater Paris area in France. The authors conducted the homeless children and families cross-sectional survey (ENFAMS: Enfants et familles sans logement) on 121 homeless sheltered mothers, who were socially disadvantaged, living in shelters in the greater Paris area in 2013, and found that 19.3% had inadequate PCU. The factors associated with PCU include socio-demographic characteristics (young age, primiparous), health status (dissatisfaction with self-perceived general health) and living conditions (housing instability in the second and third trimesters), which indicate the importance of promoting housing stability for homeless pregnant mothers, especially during PCU. The authors emphasize that housing stability for pregnant sheltered homeless mothers should be a priority to ensure better PCU and the newborn's health as much as possible.

## Intervention

In the next article, a systematic review by Ponjoan et al. looks into various published studies that assessed randomized clinical trials of COVID-19 vaccine development, and examines their reporting of age, sex, gender identity, race, ethnicity, obesity, sexual orientation, and socioeconomic status in the results that describe the participants' characteristics, loss of follow-up, stratification of efficacy and safety results. This comprehensive review of 63 articles, which assessed 20 different vaccines, mainly in phase 2 or 3, highlights the deficiency of axes of social inequity (ASI) reporting in COVID-19 vaccine trials. The ASI frequently described were age, sex and race or ethnicity, however obesity, socioeconomic status, and gender identity, sexual orientation or socioeconomic status of participants were hardly evaluated, and none of the studies included sexual orientation. The authors conclude that this lack of ASI reporting undermines their representativeness and external validity of participants and sustains health inequities.

Lastly, a prospective article by Hill et al. focuses on diversity and inclusion in clinical trials for the COVID-19 vaccine development. The authors summarize Moderna's approach toward achieving equitable representation in mRNA-1273 COVID-19 vaccine clinical trials, including the COVID-19 efficacy (COVE) study, a large, randomized, controlled, phase 3 trial of mRNA-1,273 safety and efficacy in US adults. While describing the dynamics of enrollment diversity throughout the COVE trial, the authors highlight the need for continuous, efficient monitoring and rapid pivoting from initial approaches to address early challenges and to achieve equitable representation in clinical trials. The authors further emphasize the importance of efforts toward empowering racial and ethnic minorities with the knowledge to make informed medical treatment decisions.

The Topic Editor appreciates all the authors, the reviewers, and the editorial board members for contributing to this Research Topic. We hope these women-led studies that our Research Topic brought together will inspire women researchers in publishing more as lead authors to close the gender gap and pave their path to success in the field of Life-Course Epidemiology and Social Inequalities in Health.

## Author contributions

ML has made a substantial, direct, and intellectual contribution to the work, and approved the final version of the manuscript for publication.
